# Supplementary material for: Research on the dust-control technology of a double-wall attached-ring air curtain on an excavation face
Source: PLoS One. 2024 Mar 7;19(3):e0295045. doi: 10.1371/journal.pone.0295045 (PMC10919721; doi:10.1371/journal.pone.0295045)
Supplement: S1 File — (PDF) [file pone.0295045.s001.pdf]

**Fig. 12.** The dust concentration at the X-axis section was distributed along the path at different exit speeds

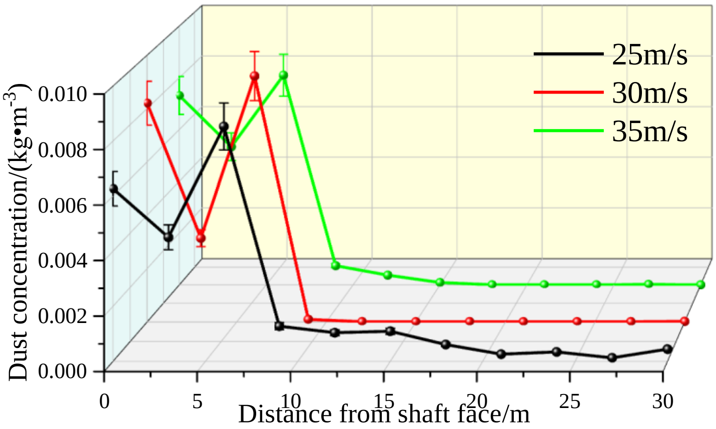


| | | | | | | |
|---|---|---|---|---|---|---|
| 0 | 0.00627 | 6.27E-04 | 0.0082 | 8.20E-04 | 0.00732 | 7.32E-04 |
| 3 | 0.0045 | 4.50E-04 | 0.00314 | 3.14E-04 | 0.00534 | 5.34E-04 |
| 6 | 0.00853 | 8.53E-04 | 0.00921 | 9.21E-04 | 0.0081 | 8.10E-04 |
| 9 | 0.00128 | 1.28E-04 | 9.50E-05 | 9.50E-06 | 7.44E-04 | 7.44E-05 |
| 12 | 0.00104 | 1.04E-04 | 1.00E-05 | 1.00E-06 | 3.77E-04 | 3.77E-05 |
| 15 | 0.00109 | 1.09E-04 | 6.00E-06 | 6.00E-07 | 9.40E-05 | 9.40E-06 |
| 18 | 6.10E-04 | 6.10E-05 | 1.00E-05 | 1.00E-06 | 6.00E-06 | 6.00E-07 |
| 21 | 2.61E-04 | 2.61E-05 | 6.90E-06 | 6.90E-07 | 6.00E-06 | 6.00E-07 |
| 24 | 3.42E-04 | 3.42E-05 | 7.00E-06 | 7.00E-07 | 6.00E-06 | 6.00E-07 |
| 27 | 1.27E-04 | 1.27E-05 | 7.00E-06 | 7.00E-07 | 2.10E-05 | 2.10E-06 |
| 30 | 4.44E-04 | 4.44E-05 | 2.10E-05 | 2.10E-06 | 6.00E-06 | 6.00E-07 |

**Fig. 17.** The dust concentration was distributed along the X-axis section using different blade attachment angles

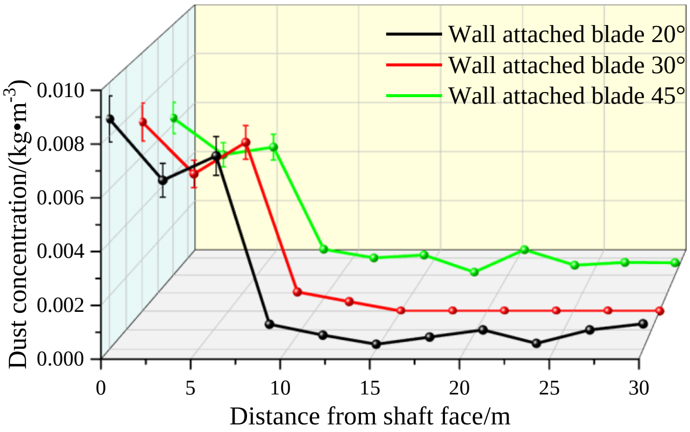


| | | | | | | |
|---|---|---|---|---|---|---|
| 0 | 0.00863 | 8.63E-04 | 0.00731 | 7.31E-04 | 0.00627 | 6.27E-04 |
| 3 | 0.00633 | 6.33E-04 | 0.00531 | 5.31E-04 | 0.0048 | 4.80E-04 |
| 6 | 0.00725 | 7.25E-04 | 0.00653 | 6.53E-04 | 0.00511 | 5.11E-04 |

| | | | | | | |
|---|---|---|---|---|---|---|
| 9 | 9.36E-04 | 9.36E-05 | 7.40E-04 | 7.40E-05 | 1.03E-03 | 1.03E-04 |
| 12 | 5.29E-04 | 5.29E-05 | 3.67E-04 | 3.67E-05 | 6.91E-04 | 6.91E-05 |
| 15 | 1.88E-04 | 1.88E-05 | 8.92E-07 | 8.92E-08 | 8.06E-04 | 8.06E-05 |
| 18 | 4.56E-04 | 4.56E-05 | 8.23E-07 | 8.23E-08 | 1.21E-04 | 1.21E-05 |
| 21 | 7.25E-04 | 7.25E-05 | 9.11E-07 | 9.11E-08 | 1.02E-03 | 1.02E-04 |
| 24 | 2.19E-04 | 2.19E-05 | 4.00E-07 | 4.00E-08 | 3.99E-04 | 3.99E-05 |
| 27 | 7.25E-04 | 7.25E-05 | 8.23E-07 | 8.23E-08 | 5.11E-04 | 5.11E-05 |
| 30 | 9.54E-04 | 9.54E-05 | 4.00E-07 | 4.00E-08 | 4.97E-04 | 4.97E-05 |
| -- | | | | | | |
| -- | | | | | | |

**Fig. 21.** Plane velocity attenuation at different positions from the wind curtain

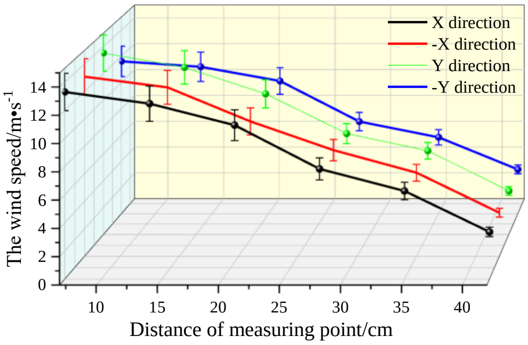


| | | | | | | | |
|---|---|---|---|---|---|---|---|
| 7 | 13.31 | 1.33E+00 | 13.08 | 1.31E+00 | 13.52 | 1.35E+00 | 11.61 | 1.161 |
| 14 | 12.48 | 1.25E+00 | 12.3 | 1.23E+00 | 12.45 | 1.25E+00 | 11.19 | 1.119 |
| 21 | 10.95 | 1.10E+00 | 9.82 | 9.82E-01 | 10.48 | 1.05E+00 | 10.11 | 1.011 |
| 28 | 7.84E+00 | 7.84E-01 | 7.73E+00 | 7.73E-01 | 7.52E+00 | 7.52E-01 | 7.02 | 0.702 |
| 35 | 6.27E+00 | 6.27E-01 | 6.09E+00 | 6.09E-01 | 6.24E+00 | 6.24E-01 | 5.82 | 0.582 |
| 42 | 3.36E+00 | 3.36E-01 | 3.17E+00 | 3.17E-01 | 3.25E+00 | 3.25E-01 | 3.37 | 0.337 |

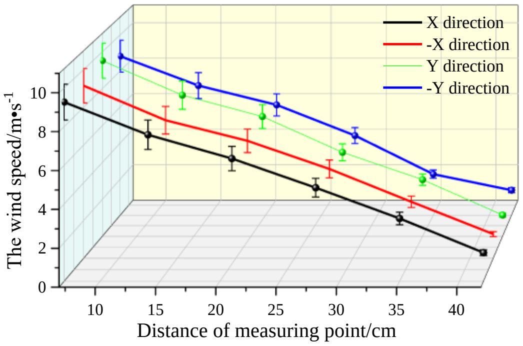


| | | | | | | | | |
|---|---|---|---|---|---|---|---|---|
| 7 | 9.27 | 9.27E-01 | 9.14 | 9.14E-01 | 9.52 | 9.52E-01 | 8.82 | 0.882 |
| 14 | 7.58 | 7.58E-01 | 7.31 | 7.31E-01 | 7.65 | 7.65E-01 | 7.19 | 0.719 |
| 21 | 6.35 | 6.35E-01 | 6.22 | 6.22E-01 | 6.49 | 6.49E-01 | 6.12 | 0.612 |
| 28 | 4.84E+00 | 4.84E-01 | 4.73E+00 | 4.73E-01 | 4.55E+00 | 4.55E-01 | 4.42 | 0.442 |
| 35 | 3.25E+00 | 3.25E-01 | 2.99E+00 | 2.99E-01 | 3.07E+00 | 3.07E-01 | 2.28 | 0.228 |
| 42 | 1.48E+00 | 1.48E-01 | 1.28E+00 | 1.28E-01 | 1.15E+00 | 1.15E-01 | 1.39 | 0.139 |

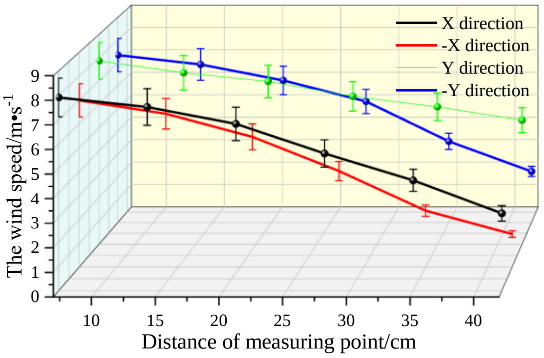


| | | | | | | | | |
|---|---|---|---|---|---|---|---|---|
| 7 | 7.91 | 7.91E-01 | 6.98 | 6.98E-01 | 7.86 | 7.86E-01 | 7.34 | 0.734 |
| 14 | 7.52 | 7.52E-01 | 6.42 | 6.42E-01 | 7.35 | 7.35E-01 | 6.93 | 0.693 |
| 21 | 6.83 | 6.83E-01 | 5.45 | 5.45E-01 | 6.98 | 6.98E-01 | 6.23 | 0.623 |
| 28 | 5.62E+00 | 5.62E-01 | 4.02E+00 | 4.02E-01 | 6.34E+00 | 6.34E-01 | 5.31 | 0.531 |
| 35 | 4.52E+00 | 4.52E-01 | 2.36E+00 | 2.36E-01 | 5.89E+00 | 5.89E-01 | 3.56 | 0.356 |
| 42 | 3.17E+00 | 3.17E-01 | 1.38E+00 | 1.38E-01 | 5.32E+00 | 5.32E-01 | 2.24 | 0.224 |

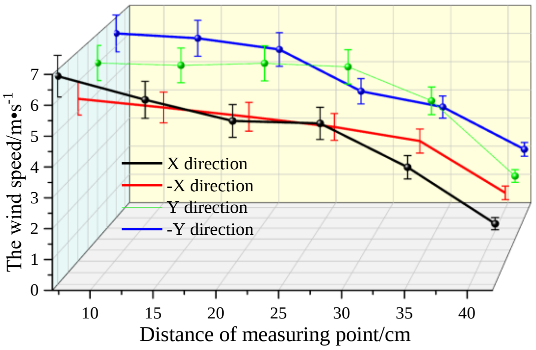


| | | | | | | | | |
|---|---|---|---|---|---|---|---|---|
| 7 | 6.79 | 6.79E-01 | 5.42 | 5.42E-01 | 6.01 | 6.01E-01 | 6.44 | 0.644 |
| 14 | 6.02 | 6.02E-01 | 5.13 | 5.13E-01 | 5.93 | 5.93E-01 | 6.27 | 0.627 |
| 21 | 5.33 | 5.33E-01 | 4.82 | 4.82E-01 | 6 | 6.00E-01 | 5.88 | 0.588 |
| 28 | 5.25E+00 | 5.25E-01 | 4.48E+00 | 4.48E-01 | 5.88E+00 | 5.88E-01 | 4.42 | 0.442 |
| 35 | 3.82E+00 | 3.82E-01 | 4.01E+00 | 4.01E-01 | 4.72E+00 | 4.72E-01 | 3.87 | 0.387 |
| 42 | 1.98E+00 | 1.98E-01 | 2.28E+00 | 2.28E-01 | 2.15E+00 | 2.15E-01 | 2.39 | 0.239 |

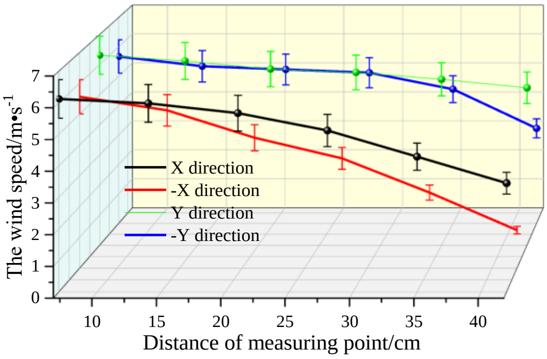


| | | | | | | | | |
|---|---|---|---|---|---|---|---|---|
| 7 | 6.12 | 6.12E-01 | 5.56 | 5.56E-01 | 6.31 | 6.31E-01 | 5.67 | 0.567 |
| 14 | 5.98 | 5.98E-01 | 5.12 | 5.12E-01 | 6.12 | 6.12E-01 | 5.34 | 0.534 |
| 21 | 5.67 | 5.67E-01 | 4.23 | 4.23E-01 | 5.85 | 5.85E-01 | 5.23 | 0.523 |
| 28 | 5.12E+00 | 5.12E-01 | 3.56E+00 | 3.56E-01 | 5.74E+00 | 5.74E-01 | 5.12 | 0.512 |
| 35 | 4.29E+00 | 4.29E-01 | 2.45E+00 | 2.45E-01 | 5.51E+00 | 5.51E-01 | 4.56 | 0.456 |
| 42 | 3.45E+00 | 3.45E-01 | 1.23E+00 | 1.23E-01 | 5.23E+00 | 5.23E-01 | 3.23 | 0.323 |

**Fig. 21.** Dust removal efficiency

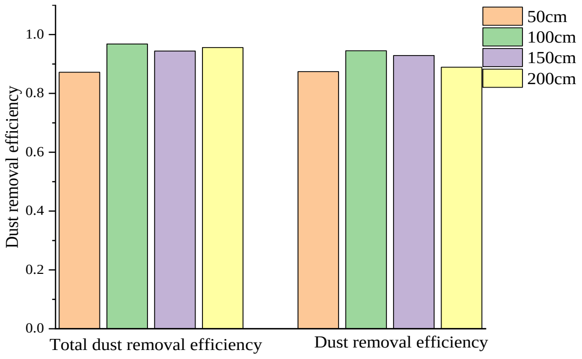


| | |
|---|---|
| 1 | 0.872 |
| 2 | 0.968 |
| 3 | 0.944 |
| 4 | 0.956 |
| 5 | 1 |
| 6 | 0.874 |
| 7 | 0.945 |
| 8 | 0.929 |
| 9 | 0.889 |

**Fig. 23.** Changes in dust concentration along the roadway

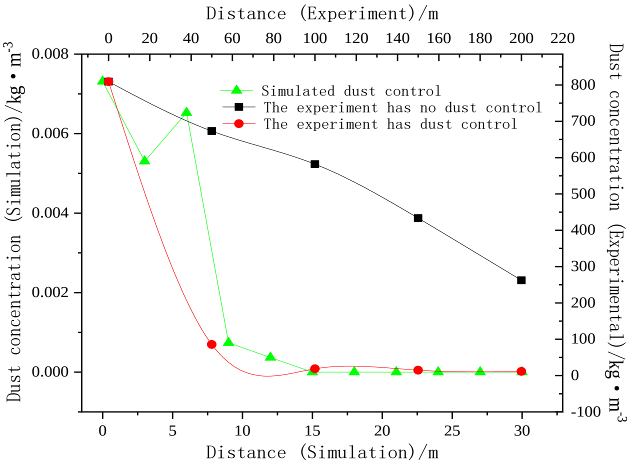


| | | | | | | |
|---|---|---|---|---|---|---|
| 0 | 0.00731 | 0.00E+00 | 809.1 | 8.09E+02 | 0 | 0.00E+00 |
| 3 | 0.00531 | 5.00E+01 | 673.3 | 8.57E+01 | -3.82E-04 | -4.17E-03 |
| 6 | 0.00653 | 1.00E+02 | 582 | 1.86E+01 | 4.38E-04 | 7.04E-04 |
| 9 | 7.40E-04 | 1.50E+02 | 4.34E+02 | 1.49E+01 | 2.66E-05 | -1.79E-04 |
| 12 | 3.67E-04 | 2.00E+02 | 2.62E+02 | 1.15E+01 | 0.00E+00 | 0.00E+00 |

| | | | | | | |
|---|---|---|---|---|---|---|
| 15 | 8.92E-07 | | | | -- | -- |
| 18 | 8.23E-07 | | | | -- | -- |
| 21 | 9.11E-07 | | | | -- | -- |
| 24 | 4.00E-07 | | | | -- | -- |
| 27 | 8.23E-07 | | | | -- | -- |
| 30 | 4.00E-07 | | | | -- | -- |
