# Supplementary material for: Research on the dust-control technology of a double-wall attached-ring air curtain on an excavation face
Source: PLoS One. 2024 Mar 7;19(3):e0295045. doi: 10.1371/journal.pone.0295045 (PMC10919721; doi:10.1371/journal.pone.0295045)
Supplement: Attachment — Submitted filename: Response to Reviewers Reviewer 3.docx [file pone.0295045.s002.docx]

Dear external audit expert, hello. Thank you for your review of my article. I will make timely revisions according to your suggestions.

(1) I will add some references according to your requirements to make the article more convincing, thank you.

(2) I have added error bars to the image data

(3) The blade Angle selected according to the real situation is: 20°, 30°, 45°, and larger or smaller angles are not in line with the reality.

(4) It has been added in the first sentence after the title of Chapter 3.

(5) It has been modified according to your comments.

Ok, thank you for your suggestion.
